# Concurrent Hypofractionated Radiotherapy and 5-Fluorouracil for Advanced Sarcomas of the Bone

**DOI:** 10.1080/13577149878127

**Published:** 1998-03

**Authors:** Charalambos Zambatis, John Skarlatos, Michael Koukourakis, Lambrini Kosma, Alexandra Giatromanolaki, Kostantinos Beroukas, Dimitrios Yannakakis

**Affiliations:** 1 Department of Radiotherapy and Oncology Hellenic Cancer Institute Saint Savvas Hospital Athens Greece; 2 Department of Radiotherapy and Oncology University Hospital of Iraklion Crete Greece; 3 Histopathology Unit Saint Nikolas General Hospital Crete Greece; 4 18 Dimokratias Avenue Crete Iraklion 71306 Greece

## Abstract

*Purpose.* 5-Fluorouracil (5-FU) has shown radiosensitizing properties *in vitro.* This paper reports the effects of radiotherapy and concomitant intravenous 5-FU radiosensitization in the treatment of advanced bone sarcomas.

*Subjects/methods.* Four patients with large inoperable bone sarcomas (three chondrosarcomas and one fibrosarcoma) were treated with hypofractionated radiotherapy and concomitant 5-FU bolus injection (300 mg m^−2^) before each fraction of radiotherapy. A radiation fraction of 5 Gy was given twice a week to a normalized total dose (α/β=4 Gy) of 75 Gy.

*Results.* The regimen was well tolerated, the main toxicity being grade I/II diarrhoea in two cases with pelvic irradiation. Treatment interruption for 1 week was necessary in two cases with pelvic disease but not in two patients treated for sarcoma of the extremities. A complete symptomatic relief was obtained in all cases immediately after the third to the fifth fraction and the median duration was 10 months. Computed tomography scan documented a partial response in 2/4 cases.

*Discussion.* Hypofractionated radiotherapy combined with potential lethal damage inhibitors for bone sarcomas requires further investigation.


*Sarcoma* (1998) **2**, 25–28

ORIGINAL ARTICLE

### Concurrent hypofractionated radiotherapy and 5-fluorouracil for advanced sarcomas of the bone

CHARALAMBOS ZAMBATIS,[1] JOHN SKARLATOS,[1] MICHAEL KOUKOURAKIS,[2] LAMBRINI KOSMA,[1] ALEXANDRA GIATROMANOLAKI,[3] KOSTANTINOS BEROUKAS[1] & DIMITRIOS YANNAKAKIS[1]

[1]*Department of Radiotherapy and Oncology, Hellenic Cancer Institute, Saint Savvas Hospital, Athens,* [2]*Department of Radiotherapy and Oncology, University Hospital of Iraklion, Crete &* [3]*Histopathology Unit, Saint Nikolas General Hospital, Crete, Greece*

**Abstract**
*Purpose.* 5-Fluorouracil (5-FU) has shown radiosensitizing properties *in vitro*. This paper reports the effects of radiotherapy and concomitant intravenous 5-FU radiosensitization in the treatment of advanced bone sarcomas.
*Subjects/methods.* Four patients with large inoperable bone sarcomas (three chondrosarcomas and one fibrosarcoma) were treated with hypofractionated radiotherapy and concomitant 5-FU bolus injection (300 mg $m^{-2}$) before each fraction of radiotherapy. A radiation fraction of 5 Gy was given twice a week to a normalized total dose ($\alpha/\beta = 4$ Gy) of 75 Gy.
*Results.* The regimen was well tolerated, the main toxicity being grade I/II diarrhoea in two cases with pelvic irradiation. Treatment interruption for 1 week was necessary in two cases with pelvic disease but not in two patients treated for sarcoma of the extremities. A complete symptomatic relief was obtained in all cases immediately after the third to the fifth fraction and the median duration was 10 months. Computed tomography scan documented a partial response in 2/4 cases.
*Discussion.* Hypofractionated radiotherapy combined with potential lethal damage inhibitors for bone sarcomas requires further investigation.

**Key words:** *bone sarcoma, hypofractionation, radiotherapy, 5-fluorouracil*

### Introduction

Sarcomas of the bone are considered to be radioresistant tumours. The only curative therapy is surgical resection.[1,2] The role of radical or pre-operative radiotherapy has been evaluated by several studies showing a 5-year disease-free survival of 20–40%.[3,4] Post-operative chemotherapy has also been used in order to enhance local control and decrease the distant metastases rate although there is no conclusive evidence of any beneficial effect.[5] Good results have been reported for tumours in axial sites that are considered inoperable and are treated with radiotherapy or combined radio-chemotherapy.[6,7]

Although 5-fluorouracil (5-FU) is not effective in bone sarcomas,[8] it has shown radiosensitizing properties *in vitro*, probably by inhibiting the radiation-induced DNA damage repair.[9] Here, we report preliminary results on four locally advanced bone sarcomas treated with high dose per fraction radiotherapy and concomitant intravenous 5-FU radiosensitization.

### Subjects and methods

### *Subjects*

Four patients with locally far advanced bone sarcomas were treated with high-dose hypofractionated radiotherapy and concommitant 5-FU chemotherapy. All patients underwent a detailed clinical and laboratory investigation (including chest and abdomen computed tomography (CT) scan). The patient and tumour characteristics are reported in Table 1. Two patients refused amputation, two had inoperable tumour (pelvic location). Three out of four patients had tumour unresponsive to adriamycin/ifosfamide chemotherapy. The age of the patients ranged from 31 to 58 years and the performance status ranged from 2 to 3. The median tumour dimensions were $13 \times 13$ cm. Three were histologically confirmed as chondrosarcomas and one as fibrosarcoma, the grade being III for all cases.

Correspondence to M. Koukourakis, 18 Dimokratias Avenue, Iraklion 71306, Crete, Greece. Tel: +30 81 392498, 284661; Fax: +30 81 392848; E-mail: TARG@HER.FORTHNET.GR.

1357-714X/98/040025-04 ©1998 Carfax Publishing Ltd

**Table 1.** *Patients and tumour characteristics*

| No. patients | sex | Age (years) | Histology | Grade | Location | Size (cm) | Previous surgery | Previous chemotherapy |
|---|---|---|---|---|---|---|---|---|
| 1 | F | 31 | ChondroSA | III | Iliac | 15 × 14 | Inoperable | Adria/Ifo |
| 2 | M | 50 | ChondroSA | III | Tibia | 12 × 10 | Refused | Adria/Ifo |
| 3 | M | 58 | ChondroSA | III | Tibia | 12 × 10 | Refused | Adria/Ifo |
| 10 | F | 45 | FibroSA | III | Iliac | 20 × 18 | Inoperable | No |

Adria = Adiamycin, Ifo = Ifosfamide.

**Table 2.** *Treatment characteristics and response in four locally advanced bone sarcomas. All patients received two 5 Gy radiotherapy fractions per week and 300 mg m$^2$ of 5-FU intravenously before each fraction*

| No. patients | Dose Fraction (Gy) × no. fractions | NTD (Gy, a/b = 4) | Symptomatic relief | Response rate | Duration of response (months) | Overall survival (months) |
|---|---|---|---|---|---|---|
| 1 | 5 × 10 | 75 | SSR | PR | 15 | 15 + |
| 2 | 5 × 10 | 75 | SSR | SD | 8 | 14 (l,b) |
| 3 | 5 × 10 | 75 | SSR | MR | 12 | 12 + |
| 10 | 5 × 10 | 75 | SSR | MR | 6 | 6 (pe) |

NTD = Normalized total dose, SSR = substantial symptomatic relief, PR = bidimensional measurements of the tumour reduced by > 50%, MR = reduced by 20–50%, PD = progessive disease, + = still alive, l/b = death from lung/bone metastases, oe = death from pulmonary embolus.

*Treatment*

The treatment characteristics are reported in Table 2. All cases were treated with a 6 MV X-ray linear accelerator and, where feasible, part of the dose was given with 15–25 MeV electrons. A CT scan-based radiotherapy treatment planning was considered for all cases; 5 Gy were given per fraction twice a week for a total number of 10 fractions. The normalized total dose (NTD)[10.11] to both the tumour and normal tissue was calculated for $\alpha/\beta = 4$ Gy, although higher values of $\alpha/\beta$ ratio (4–10 Gy) have been reported from radioresponsiveness experiments in sarcoma cell lines.[12] The tumour NTD was 75 Gy. An intravenous bolus dose of 300 mg/m$^2$ of 5-FU was given 1 h before each fraction of radiotherapy. Metoclopramide 10 mg was given intravenously before the injection of 5-FU.

Assessment of acute toxicity followed the WHO toxicity scale.[13] Full blood count and biochemical tests were done weekly. Performance status was assessed every 2 weeks. Assessment of response was done clinically and with CT scan 4 weeks after the completion of treatment and 4-monthly thereafter. Patients were followed bimonthly with clinical examination, blood tests and chest X-ray.

### Results

No severe haematological or organ-specific acute toxicity was observed. Grade I/II skin desquamation was also observed in all four patients. Grade I haematological toxicity (neutropenia and/or anaemia) was observed in 2/4 cases. Diarrhoea grade I appeared in two patients with pelvic disease and was well controlled with oral medication. In these two cases the treatment was interrupted for 1 week. No fibrosis or late sequel has been observed (6–18 months after radiotherapy.

The main symptomatology of all four treated cases was uncontrollable pain and three patients were under heavy analgesic medication with morphine. Substantial pain relief was obtained in all four patients immediately after the third to fifth fraction and morphine was discontinued. The median duration of symptomatic control was 10 months. CT scan confirmed a partial response (50–95% reduction in two-dimensional measurement) in 2/4 (50%) cases. Figure 1(a) and 1(b) shows a case of chondrosarcoma of the iliac bone with partial response 6 months after treatment.

### Discussion

Bone sarcomas other than Ewing's sarcoma are considered to be relatively radioresistant tumours in which radical treatment is achieved only when surgery is applied with or without adjuvant radiotherapy or chemotherapy. In a recent study from the Norwegian Radium Hospital,[14] complete tumour removal was a major prognostic factor. Unfortunately, surgery cannot be applied in bone sarcomas of the axial skeleton, and pelvic tumour location requires extensive amputation with severe impact on the quality of life. In these cases, curative radiotherapy should be tried as an alternative to surgery. A 40% 5-year actuarial survival has been reported by Krochak *et al.*[3] on 38 chondrosarcomas of bone, treated with conventionally fractionated radiother-

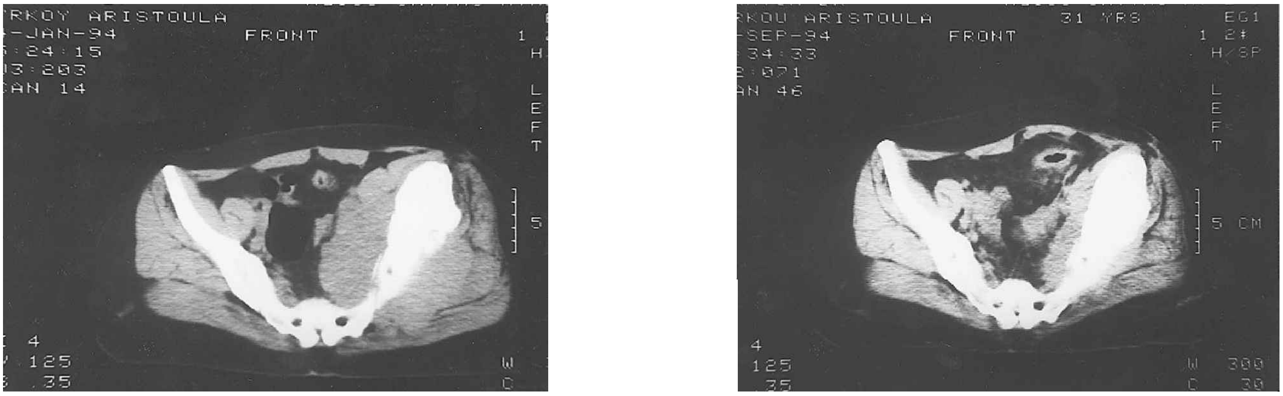

*(a)* *(b)*

**Fig. 1.** *A large chondrosarcoma of the iliac bone (a) in a 31-year-old woman treated with 10 fractions of 5 Gy (two fractions per week) and concurrent 300 mg/m*$^2$ *5-FU bolus injection 1 h before radiotherapy. Bidimensional measurements 6 months after the end of radiatherapy showed 90% reduction of tumour size (b). The patient is alive and disease free 15 months after completion of radiotherapy.*

apy. However, tumour size has not been analyzed and patients with high-grade tumour had a 5-year survival of 22%.

In the present study, we treated four patients with locally far advanced high-grade bone sarcomas with radical radiotherapy. In order to increase the results of radiotherapy, we used hypofractionation combined with 5-FU radiosensitization. The NTD was 75 Gy. Partial response was observed in 2/4 patients. Kinsella *et al.*[7] reported a study on 10 unresectable bone and soft tissue sarcomas treated with large fraction radiotherapy and misonidasole radiosensitization. Although no complete remission was observed, stabilization of the disease and complete symptomatic relief was achieved in 7/10 patients. This is in accordance with our results where a long lasting (>6 months) symptomatic relief was obtained in all four cases. In a recent study, Hug *et al.*[6] treated seven cases of bone tumours of the axial skeleton with definitive radiotherapy using a combination of protons and photons. Local failure was observed in 2/7 cases showing that a high radiation dose results in good local control. A high local control rate (7/9 patients) has also been reported by the Standford Group[15] where intra-arterial 5-bromodeoxyuridine was given together with hypofractionated radiotherapy for osteosarcomas. However, extensive local tissue toxicity was observed, including subcutaneous fibrosis, neuropathy and non-healing traumas.

The optimal way to treat inoperable sarcomas has not yet been defined. It seems that high-dose radiotherapy offers excellent symptomatic relief and results in long-term progression-free survival in about 20% of cases. Distant metastases remain a main cause of failure in high-grade bone sarcomas. Our preliminary experience shows that hypofractionated radiotherapy for bone sarcomas combined with potential lethal damage inhibitors may have a role in the control of local disease.

### References

1 Simon MA, Aschliman MA, Thomas N, *et al.* Limb-salvage treatment versus amputation for osteosarcoma of the distant end of the femur. *J Bone Joint Surg* 1986; 68:1331–7.
2 Pritchard DJ, Lunke RJ, Taylor WF, *et al.* Chondrosarcoma: a clinicopathologic statistical analysis. *Cancer* 1980; 45:149–57.
3 Krochak R, Harwood AR, Cummings BJ, *et al.* Results of radical radiation for chondrosarcoma of bone. *RadiotherOncol* 1983; 1:109–15
4 Cade S. Osteogenic sarcoma: a study based on 133 patients. *J R Coll Surg Edinb* 1955; 1:79–111.
5 Provisor A, Nachman J, Krailo M, *et al.* Treatment of non metastatic osteogenic sarcoma of the extremeties with pre and post-operative chemotherapy. *Proc Am Soc Clin Oncol* 1987; 6:217.
6 Hug EB, Fitzek MM, Liebsch NJ, *et al.* Locally challenging osteo and chondrogenic tumours of the axial skeleton: results of combined proton and photon radiation radiation therapy using three-dimensional treatment planning. *Int J Radiat Oncol Biol Phys* 1995; 31:467–76.
7 Kinsella TJ, Glatstein E. Clinical experience with intravenous radiosensitizers in unresectable sarcomas. *Cancer* 1987; 59:908–15.
8 Pratt CB, Meyer WH, Howlett N *et al.* Phase II study of 5-fluorouracilucovorin for pediatric patients with malignant tumours. *Cancer* 1994; 74:2593–8.
9 Hughes LL, Luengas J, Rich TA, *et al.* Radiosensitization of cultured human colon adenocarcinoma cells by 5-flourouracil: effects on cell survival, DNA repair and cell recovery. *Int J Radiat Oncol Biol Phys* 1992; 23:983–91.
10 Macejewski B, Taylor JM, Wither HR. Alpha/beta and the importance of the size of dose per fraction for late complications in the supraglottic larynx. *Radiother Oncol* 1986; 7:323–6.
11 Koukourakis M, Damilakis J. LQ-based model for biological radiotherapy planning. *Med Dosim* 1994; 19:269–77.
12 Stuschke M, Volker B, Sack H. Radioresponsiveness of human glioma, sarcoma and breast cancer spheroids depends on tumour differentiation. *Int J Radiat Oncol Biol Phys* 1993; 27:627–36.
13 World Health Organisation. *Handbook for reporting results of cancer treatment*. Geneva: WHO Offset Publ., 1979; 48.
14 Saeter G, Hoie J, Stenwig A, *et al.* Systemic relapse of patients with osteogenic sarcoma. *Cancer* 1995; 75:1084–93.

15 Martinez A, Goffinet DR, Donaldson SS, *et al*. Intra-arterial infusion of radiosensitizer (BUdR) combined with hypofractionated irradiation and chemotherapy for primary treatment of osteogenic sarcoma. *Int J Radiat Oncol Biol Phys* 1985; 11:123–8.
